# What works and for whom? Individual patient data meta-analyses in global nutrition research

**DOI:** 10.1093/ajcn/nqab316

**Published:** 2021-09-29

**Authors:** Christopher R Sudfeld, Emily R Smith

**Affiliations:** Department of Global Health and Population, Harvard T.H. Chan School of Public Health, Boston, MA, USA; Department of Nutrition, Harvard T.H. Chan School of Public Health, Boston, MA, USA; Department of Global Health, Milken Institute School of Public Health, The George Washington University, Washington, DC, USA; Department of Exercise and Nutrition Sciences, Milken Institute School of Public Health, The George Washington University, Washington, DC, USA

The seminal paper by Bhutta et al. in the 2008 Lancet Maternal and Child Undernutrition Series addressed the question “What works?” to improve maternal and child survival, health, and nutrition (1). Since then, we have expanded our toolkit of evidence-based interventions that improve child health, growth, and development, including small-quantity lipid-based nutrient supplementation (SQ-LNS) for children (2). Concurrently, there has also been a growing appreciation that the effects of these interventions may differ for individuals and populations. Effect modification or heterogeneity in the magnitude of impact of nutrition interventions in subpopulations of participants with different characteristics may be attributable to various mechanisms such as those related to baseline nutritional deficiency, environmental factors that affect nutrient absorption and storage, sex-specific effects, genetics, and others (3, 4). This concept has also been more broadly recognized through the emergence of “precision public health,” which focuses on “providing the right intervention to the right population at the right time” (5). Accordingly, there is a growing global nutrition literature, including this companion supplement entitled “Lipid-Based Nutrient Supplements,” that expands the original question asked by Bhutta and colleagues to What works *and* for whom?

Individual patient data (IPD) meta-analyses are considered the “gold standard” for evidence synthesis and analyze original participant data from multiple studies with a consistent quality assessment and statistical approach (6, 7). IPD meta-analyses offer multiple advantages over aggregate study-level data meta-analyses, including greater statistical power and a harmonized approach to assess effect modification (6, 7). Therefore, IPD meta-analyses offer the most robust approach to answer questions about who benefits from interventions. The IPD meta-analyses of child SQ-LNS published in this supplement identified several effect modifiers that would not have been identifiable in study-level analyses—there was a greater effect of child SQ-LNS on stunting, wasting, and other anthropometric measures for girls as compared with boys; on language, motor, and executive function for children in households with low socioeconomic status; and on anemia among children in households with improved water quality (8–11).

Effect modification findings from IPD meta-analyses can give important insight into biological mechanisms, as well as have important program and policy implications. While it is clear based on the aggregate data meta-analysis that child SQ-LNS provided overall beneficial effects on child growth, development, and micronutrient status, the IPD meta-analysis suggests that we should expect the magnitude of SQ-LNS program impacts to vary depending on characteristics of the target population and the outcome of interest (8). Further, programs can also determine the need for targeting interventions to specific populations, address inequalities, or optimize resource utilization given constraints (12). For example, based on the new IPD meta-analyses, a social protection program may consider targeting low-socioeconomic-status households with child SQ-LNS to improve development outcomes (10). In addition, IPD meta-analyses can also address whether subpopulations may experience safety risks. For example, we conducted an IPD meta-analysis on multiple micronutrient supplementation (MMS) in pregnancy that addressed concerns on the safety of MMS for women with short stature; we provided direct evidence that there was no increased risk of adverse outcomes for short stature or any subpopulation of pregnant women (13).

Despite the many strengths of IPD meta-analyses, there are important logistic and methodological limitations to the study design. First, obtaining IPD from each study can be challenging because some investigators may be unwilling or unable to share individual-level data. It is possible that investigators could analyze their own data with a unified statistical approach to provide effect estimates if a 2-stage IPD meta-analytic approach is used. However, while 1-stage (individual-level data pooled in a single dataset and analyzed) and 2-stage approaches for IPD meta-analyses will give the same results when the same modeling assumptions are made, the 1-stage approach offers additional modeling flexibility (14). Further, while IPD meta-analyses allow more thorough investigations of outcomes and subgroups, these analyses also suffer from limitations associated with multiple testing. In the case of investigating heterogeneity, the statistical issue is compounded by the examinations of multiple correlated outcomes across multiple correlated subpopulations of interest. For example, in the SQ-LNS IPD meta-analysis on child development outcomes, there were 6 continuous development domain outcomes analyzed by 16 subgroup effect modifiers, which led to 96 statistical tests, of which 13 *P* values for effect modification were significant at the *P* < 0.10 level (10). If these tests were fully independent, we would expect ∼9 *P* values <0.10 by chance alone; however, we know that, in this case, the outcomes and modifiers in the analysis were correlated to some degree. Therefore, it is probable that there are false discoveries among the effect modifiers identified in the IPD meta-analyses and advanced statistical methods would be needed to directly address multiple testing of correlated outcomes (15). Regardless, effect modifiers and outcomes should be chosen a priori based on biological plausibility and implementation plans. The possibility of false discovery should be considered an important, if not inherent, limitation to IPD meta-analyses that evaluate multiple outcomes and potential effect modifiers.

Further, there are several methodological innovations that can provide opportunities to ensure timely evidence synthesis. A prospective meta-analysis identifies studies that will contribute data to the meta-analysis, as well as establishes the logistical and methodological plans for an eventual IPD meta-analysis, before the results of the individual studies are known (16). Tierney and colleagues (17) propose a framework for prospective, adaptive meta-analysis of aggregate data for faster, collaborative evidence synthesis for existing and ongoing trials. There are also methods for sequential or cumulative meta-analyses that focus on identifying when meta-analyses should be updated and when there are enough data for a meta-analysis to provide meaningful results that can be applied to IPD meta-analyses (16). A common thread across all approaches is that developing a rigorous plan and protocol for pooling data and statistical analysis is essential for the rigorous conduct of an IPD meta-analysis; this SQ-LNS supplement serves as model for the process of planning, documentation, and implementation of an IPD meta-analysis.

Given the importance of IPD meta-analyses, both individual study investigators and funders should plan for data pooling before starting new studies to ensure that the design, interventions and comparisons groups, outcome assessments, and data are collected and available in a way that allows for conduct of an IPD meta-analysis. Plans for data-sharing can also then be appropriately considered in participant consent forms, ethical approvals, and data use agreements from the beginning of studies. As a global nutrition community, we must recognize the importance of proactive planning and commitment to collaborative IPD meta-analyses to address what works and for whom to inform effective programs and policies in the context of global inequalities, continuing resource constraints, and growing interest in precision public health.

## Notes

The authors reported no funding received for this study.
